# Combined Effect of DNase I and Proteinase K on Dual‐Species Biofilm of *Campylobacter jejuni* and *Acinetobacter baumannii*

**DOI:** 10.1155/ijfo/6948459

**Published:** 2025-12-02

**Authors:** Joo Young Lee, Joo-Sung Kim

**Affiliations:** ^1^ Korea Food Research Institute, Wanju-gun, Republic of Korea, kfri.re.kr; ^2^ Department of Food Science and Biotechnology, Sungkyunkwan University, Suwon-si, Republic of Korea, skku.edu; ^3^ Department of Food Biotechnology, Korea University of Science and Technology, Daejeon, Republic of Korea, ust.ac.kr

**Keywords:** biofilm, *Campylobacter*, DNase I, dual-species, proteinase K, reduction

## Abstract

In food‐associated environments, foodborne bacteria can form biofilms that are likely to exist as multiple species and are potentially a food safety concern. In this study, we focused on the effects of DNase I and proteinase K on dual‐species biofilms containing *Campylobacter jejuni* and bacterial isolates from food‐associated environments. The viable cell counts of *C. jejuni* differed significantly depending on the counterpart strain in the biofilms. In mature monospecies biofilms, both *C. jejuni* and *Acinetobacter baumannii* were susceptible to both enzymes. *Acinetobacter baylyi* was susceptible only to DNase I, while *Staphylococcus epidermidis* was susceptible only to proteinase K. Analysis of confocal laser scanning microscopy images of *A. baumannii* biofilm showed that the protein distribution was consistent with that of the biofilm‐embedded cells, whereas it was distinct from the polysaccharide distribution. Among the dual‐species biofilms, that of *C. jejuni* and *A. baumannii* was the only biofilm susceptible to both enzymes. Combined treatment using DNase I followed by proteinase K was far more effective than DNase I monotherapy against both *A. baumannii* mono‐ and dual‐species biofilms. Our study suggests that proteins could be a primary target for inactivating biofilm‐embedded cells in *A. baumannii*, and the use of multiple enzymes could be an efficient strategy for biofilm removal.

## 1. Introduction

*Campylobacter* is one of the most important foodborne pathogens that cause foodborne illnesses worldwide. It was estimated to account for approximately 100 million cases of foodborne illness, with 21,000 deaths recorded worldwide in 2010 [1]. The growth requirements of *Campylobacter* are unique because it is microaerophilic and can grow at temperatures above 30°C. Among the *Campylobacter* species, *Campylobacter jejuni* is responsible for most cases of campylobacteriosis in humans.

*Acinetobacter baumannii* is a gram‐negative opportunistic pathogen that has emerged as a critical multidrug‐resistant (MDR) organism of global concern. As a member of the ESKAPE group of nosocomial pathogens, *A. baumannii* is a major contributor to healthcare‐associated infections, demonstrating exceptional resistance to antibiotics and persistence in hospital environments [2]. Although historically regarded as primarily a healthcare‐associated pathogen, emerging evidence suggests that *A. baumannii* is increasingly being isolated from food, raising concerns about its potential role as a foodborne pathogen [3, 4]. Recent studies have identified the presence of MDR *A. baumannii* strains across various food types, including fresh produce, meat products, dairy, and seafood; some of these strains exhibit significant genetic homology to clinically derived strains, suggesting potential transmission pathways between environmental, food, and healthcare settings [5–8]. These findings underscore the necessity for further investigation of the prevalence, transmission dynamics, and public health implications of *A. baumannii* in a food‐related setting.

Bacterial biofilms are dynamic matrices in which bacterial cells are embedded in an extracellular matrix, which usually comprises self‐secreted proteins, polysaccharides, extracellular DNA (eDNA), and lipids [9, 10]. Biofilms can be exploited as a strategy for bacteria to survive in hostile environments such as extreme temperatures and desiccation and the presence of salts, sanitizers, and antimicrobials [9–12]. Like many bacterial species, *Campylobacter* is able to form biofilms on abiotic surfaces including food contact surfaces [13]. Kitchen surfaces, including cutting boards, knives, and work surfaces, can be contaminated with *Campylobacter*, which can be a source of cross‐contamination of food materials, eventually leading to human infections [14]. Biofilms formed by *Campylobacter* on such surfaces are a potential food safety concern. Therefore, it is important to understand *Campylobacter* biofilms and develop effective control measures.

Bacterial biofilms formed on surfaces are likely to exist in multiple species rather than as monospecies [15, 16]. Although *Campylobacter* has been confirmed to form mixed‐species biofilms with other bacteria, including *Escherichia coli*, *Staphylococcus aureus*, and *Pseudomonas aeruginosa* [17–20], *Campylobacter*‐containing mixed‐species biofilm formation remains poorly understood [21].

eDNA and proteins comprise bacterial biofilms and can be effective targets for biofilm control [22–25]. *Campylobacter* biofilms also contain eDNA and proteins in extracellular matrices and have been demonstrated to be effectively controlled or degraded by DNase I and proteinase K treatment [22, 25–27]. However, the efficacy of DNase I and proteinase K treatments against *Campylobacter*‐containing mixed‐species biofilms remains poorly understood. Therefore, we attempted to understand the biofilm formation of *C. jejuni* NCTC11168 mixed with food environment–associated contaminating microorganisms to assess the potential application of DNase I and proteinase K in mitigating biofilm formation.

## 2. Materials and Methods

### 2.1. Bacterial Strains

The bacterial strains used in this study are listed in Table 1. *C. jejuni* NCTC11168 was purchased from the American Type Culture Collection (Manassas, VA, United States), and the bacterial strains used in mixed‐species biofilms were originally isolated from a cafeteria kitchen [28].

**Table 1:** Bacterial strains used in this study.

**Strains** ^**a**^	**Sources**
*Campylobacter jejuni* NCTC11168	American Type Culture Collection
*Acinetobacter baylyi* 9‐4	Countertop for completed menu
*Acinetobacter baumannii* 9‐18	Countertop for completed menu
*Bacillus licheniformis* 5‐14	Roasting/frying countertop area
*Bacillus subtilis* 8‐3	Frying pot
*Kocuria kristinae* 5‐3	Roasting/frying countertop area
*Staphylococcus epidermidis* 11‐2	Food waste container

^a^All strains were isolated from a cafeteria kitchen in a previous study, except for *C. jejuni* [28].

### 2.2. Culture Conditions and Preparation of Inoculum

The inoculum of *C. jejuni* was prepared as previously described, with minor modifications [29]. NCTC11168 strain in glycerol stock at −80°C was inoculated onto tryptic soy agar supplemented with 5% (*v*/*v*) sheep blood (TSAB) and incubated at 37°C for 2 days under microaerobic conditions (6%–12% *v*/*v* O_2_ and 5%–8% *v*/*v* CO_2_) using an AnaeroPack‐MicroAero (Mitsubishi Gas Chemical Co., Tokyo, Japan). Then, the cells were suspended in sterile Mueller–Hinton broth (MHB) at OD_600_ of 0.1–0.2 measured by a BioPhotometer plus spectrophotometer (Eppendorf, Hamburg, Germany). Suspended cells (100 *μ*L) were spread on TSAB and incubated overnight (approximately 16 h) at 37°C under microaerobic conditions. Overnight cultures were resuspended in MHB, and the OD_600_ was adjusted to 0.005–0.014 using MHB for inoculation.

To prepare the inocula of contaminating microorganisms derived from a cafeteria kitchen [28], 5 mL MHB in each sterile tube was inoculated with each strain grown on TSAB or strain‐containing porous beads of Microbank (Pro‐Lab Diagnostics, Round Rock, TX, United States) directly from −80°C storage. Then, the inoculated tubes were incubated at 38°C with shaking at 150 rpm overnight. Overnight cultures were diluted in MHB to an OD_600_ of 0.005–0.014 for inoculation.

### 2.3. Mixed‐Species Biofilm Formation

The inocula of contaminating microorganisms were mixed with an equal volume of *C. jejuni* inoculum for mixed‐species biofilm formation. A sample (100 *μ*L) of the mixed‐species or monospecies cultures was added to each well of a polystyrene 96‐well microtiter plate (SPL Life Science, Seoul, Republic of Korea). The plates were incubated at 37°C under microaerobic conditions for 48 h.

### 2.4. Viable Cell Counts

To quantify viable cells in biofilms, the media were decanted from the biofilm‐grown wells, and each well was washed in 150 *μ*L of sterile 0.85% NaCl solution by pipetting up and down four times [29]. After another washing, 100 *μ*L of sterile 0.85% NaCl solution and three small beads (3 mm in diameter; Glastechnique Mfg., Germany) were added to each well. The microtiter plate was shaken at 1000 rpm for 10 min using ThermoMixer C (Eppendorf). Then, the liquid was transferred to 0.9 mL of sterile 0.85% NaCl solution, and the remaining biofilms on the plate surface were scraped off with pipette tips, followed by vortexing. The cell suspension was serially diluted 10‐fold in sterile 0.85% NaCl solution, and 100 *μ*L of each diluent was spread on TSAB and/or Preston agar [27]. The spread TSAB and Preston agar plates were incubated at 37°C under aerobic conditions for 24 h to grow contaminating bacteria and under microaerobic conditions for 48 h to grow *Campylobacter*, respectively. Preston agar was prepared by supplementing Campylobacter Agar Base (Oxoid, Basingstoke, England) with laked horse blood (5%), polymyxin B (5 IU/mL), rifampicin (10 *μ*g/mL), trimethoprim (10 *μ*g/mL), and amphotericin B (10 *μ*g/mL) (Oxoid) [27]. The colonies were then counted, and the colony‐forming units (CFUs) per well were calculated based on the dilution factor.

### 2.5. DNase I Treatment

The medium in the biofilm‐grown wells was replaced with 100 *μ*L fresh MHB and DNase I (Thermo Fisher Scientific, Waltham, MA, United States) was added at a final concentration of 0.1 U/100 *μ*L MHB as previously described [27]. The plates were incubated aerobically at 37°C for 1 h.

### 2.6. Proteinase K Treatment

The culture medium in the biofilm‐containing wells was replaced with 100 *μ*L of fresh MHB. Proteinase K (Qiagen, Hilden, Germany) was added to achieve a final concentration of 10 mAU/mL in MHB [27]. The plates were then incubated under aerobic conditions at 37°C for 1 h.

### 2.7. Combined Treatment Using Proteinase K With DNase I

To assess the synergistic effect of enzyme treatment on biofilm removal, both mono‐ and dual‐species biofilms were exposed to DNase I and proteinase K.

For DNase I treatment, the supernatant was carefully removed from each well. The DNase I was administered at a concentration of 0.1 U/100 *μ*L MHB. The plates were then incubated at 37°C for 1 h. Following incubation, the wells were washed once with 150 *μ*L of MHB per well. Subsequently, proteinase K was added at a final concentration of 10 mAU/mL MHB, and the plate was incubated for an additional hour at 37°C. The wells were then washed twice with 150 *μ*L of sterile distilled water.

### 2.8. Crystal Violet Assay

The biofilm mass was quantified using crystal violet, as described previously [27]. Briefly, the medium was removed from the biofilm‐grown or enzyme‐treated wells. Then, the wells were rinsed twice with 150 *μ*L of sterile deionized water. After complete drying, 100 *μ*L of 1% crystal violet solution (Sigma‐Aldrich, St. Louis, MO, United States) was added to each well and incubated for 30 min at room temperature. The wells were then washed with slowly running tap water, gently rinsed with deionized water, and dried. The dye was solubilized in a 100 *μ*L mixture of 30% methanol and 10% acetic acid per well. The absorbance was measured at 590 nm using an Infinite M200 Pro NanoQuant microplate reader (Tecan, Männedorf, Switzerland).

### 2.9. Statistical Analysis

Data were analyzed using SPSS statistical software (SPSS Inc. IBM Co., Armonk, NY, United States). For multiple comparisons, one‐way analysis of variance (ANOVA) was used with Tukey′s honest significant difference (HSD) test. A *t*‐test was used to compare two groups (*p* < 0.05).

### 2.10. Confocal Laser Scanning Microscopy (CLSM)

To distinguish among proteins, carbohydrates, and cells within biofilms, a previously established protocol was used [30]. The cell suspension was prepared as previously described and incubated in a 96‐well plate with a transparent polymer coverslip at the base (*μ*‐plate 96‐well black; ibidi, Gräfelfing, Germany). Following enzyme treatment, each well was rinsed four times with 200 *μ*L of filter‐sterilized deionized water. Red fluorescent nucleic acid stain (SYTO 63; Life Technologies, Carlsbad, CA, United States) diluted to 20 *μ*M was added (200 *μ*L per well) and incubated at room temperature in darkness for 30 min. Subsequently, the wells were rinsed with sterile PBS for 1 min. A FITC solution (0.1 mg/mL, Sigma‐Aldrich) prepared in 0.1 M sodium bicarbonate buffer (pH 8.5–9.0) was added (200 *μ*L per well) and incubated at room temperature in darkness for 1 h. Following this, the wells were washed again with sterile PBS for 1 min, after which concanavalin A conjugated with tetramethylrhodamine (Life Technologies) diluted to 0.1 mg/mL in 0.1 M sodium bicarbonate buffer (pH 8.5–9.0; Sigma‐Aldrich) was added at 200 *μ*L per well and the cells were incubated for 30 min in darkness. Finally, the wells were rinsed twice with filter‐sterilized deionized water for 1 min per wash. Proteins, carbohydrates, and cells within the biofilms attached to the bottom surface were visualized using an LSM 880 with Airyscan (Carl Zeiss, Oberkochen, Germany) at excitation/emission wavelengths of 488 nm/500–535 nm for proteins, 561 nm/571–615 nm for carbohydrates, and 633 nm/650–758 nm for cells.

## 3. Results

To demonstrate the formation of dual‐species biofilms comprising *Campylobacter* and the bacteria commonly contaminating the food‐associated environment, *C. jejuni* NCTC11168 strain was mixed with each of the selected biofilm‐forming bacterial isolates from a cafeteria kitchen, incubated at 37°C under microaerobic conditions for 48 h, and the number of viable cells in biofilms was assessed using selective viable cell counts (Figure 1). Overall, *C. jejuni* was recovered from all tested dual‐species biofilms. The viable cell counts of *C. jejuni* differed significantly depending on the counterpart strain in the biofilms, whereas only *Bacillus licheniformis* and *Staphylococcus epidermidis* among the counterpart strains demonstrated a significant change in the viable cell counts (Figure 1). The viable cell counts of *C. jejuni* varied between 2 and 6 log CFU/well, depending on the counterpart strain. They were significantly reduced from 4–5 to 2 log CFU/well in the presence of *Acinetobacter baylyi* and to 3.5 log CFU/well in the presence of *A. baumannii* (*p* < 0.05) (Figure 1a), whereas they were significantly increased to 6 log CFU/well in the presence of *S. epidermidis* (*p* < 0.05) (Figure 1b). In addition, there was a significant but slight increase in the viable count of *C. jejuni* in the presence of *B. licheniformis* (*p* < 0.05) (Figure 1b). No significant changes were observed in the presence of the other isolates (*p* > 0.05).

Figure 1Viable cell counts of bacteria embedded in mono‐ or mixed‐species biofilms comprising *C. jejuni* and contaminating bacteria isolated from a cafeteria kitchen were obtained by mixing *Campylobacter jejuni* with (a) *Acinetobacter*, a gram‐negative bacteria, or with (b) *Bacillus*, *Kocuria*, or *Staphylococcus*, which are gram‐positive bacteria. Then, the mono‐ or dual‐species biofilms were formed on a polystyrene 96‐well microtiter plate at 37°C under microaerobic conditions for 48 h. The data are based on four independent experiments. The asterisks (∗) above the bars indicate significant differences compared to the corresponding monospecies biofilms at *p* < 0.05 using *t*‐test.

**Figure:**
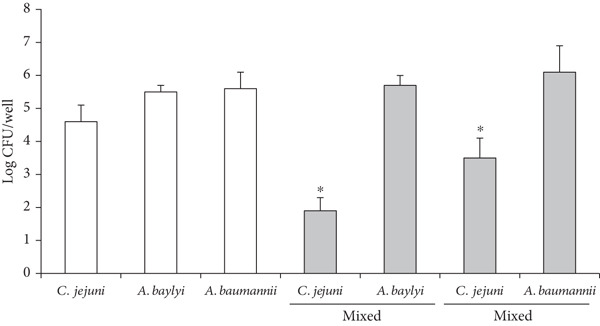
(a)

**Figure:**
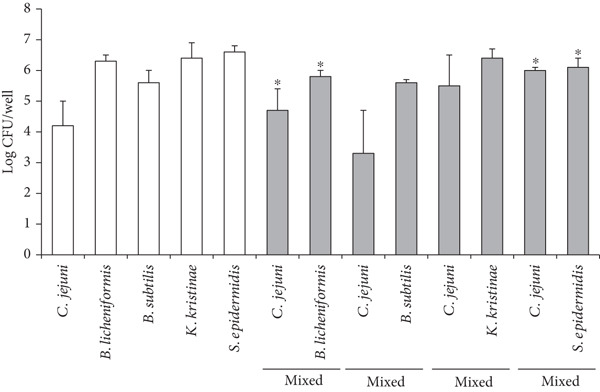
(b)

**Figure 2:**
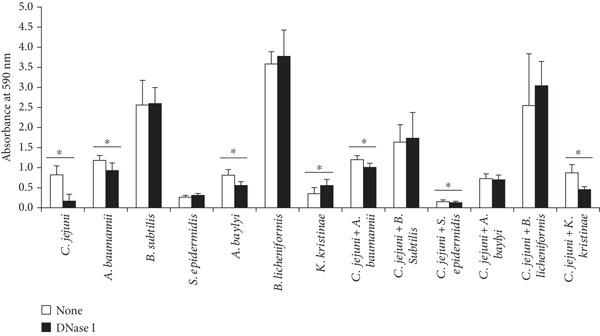
Quantification of surface‐attached mono‐ or dual‐species biofilms comprising *C. jejuni* and contaminating bacteria isolated from a cafeteria kitchen before or after DNase I treatment. Biofilms were formed on a polystyrene 96‐well microtiter plate at 37°C under microaerobic conditions for 48 h, treated with DNase I at 37°C for 1 h, and quantified using a crystal violet assay. The data are based on three independent experiments in triplicate. Asterisk (∗) denotes a significant difference between untreated and DNase‐I treated samples (*p* < 0.05).

Mono‐ or dual‐species biofilms were treated with DNase I, and their biofilm mass was measured using a crystal violet assay (Figure 2). Consistent with a previous study [26], biofilm formation by *C. jejuni* NCTC11168 was significantly reduced after DNase I treatment (*p* < 0.05). Among the monospecies biofilms of the contaminating microorganisms, the biofilm formation of *A. baumannii* and *A. baylyi* was significantly reduced (*p* < 0.05). However, *Bacillus subtilis*, *B. licheniformis*, *S. epidermidis*, and *Kocuria kristinae* were either resistant to DNase I treatment or significantly increased after DNase I treatment (*p* < 0.05) (Figure 2). In dual‐species biofilms, the biofilms of *A. baumannii*, *S*. *epidermidis*, and *K. kristinae* were significantly reduced after DNase I treatment (*p* < 0.05), whereas those of *A. baylyi*, *B. subtilis*, and *B. licheniformis* were not affected (Figure 2). Interestingly, the dual‐species biofilm containing both *C. jejuni* and *A. baylyi* was resistant to DNase I treatment, whereas each species alone was susceptible to DNase I treatment (Figure 2).

In addition, biofilm mass in the absence of DNase I treatment was compared between the dual‐species and monospecies biofilms (Figure 2). The mass of the dual‐species biofilms was lower than that of their summed monospecies counterparts for all tested biofilms. The addition of *C. jejuni* increased only the mass of the biofilm containing *K. kristinae* (Figure 2). Such a small effect of *C. jejuni* on mixed‐species biofilms was commonly observed in a previous study [19].

**Figure 3:**
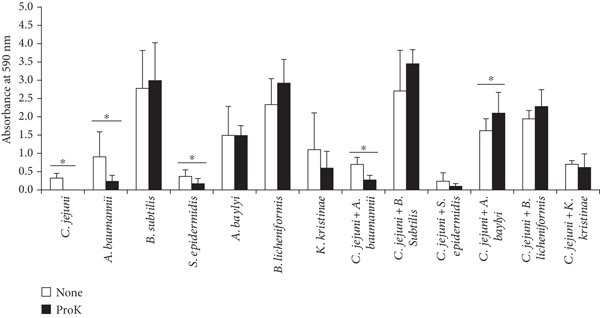
Quantification of surface‐attached biofilms of mono‐ or dual‐species biofilms comprising *C. jejuni* and contaminating bacteria isolated from a cafeteria kitchen after proteinase K treatment. Biofilms were formed on a polystyrene 96‐well microtiter plate at 37°C under microaerobic conditions for 48 h, treated with proteinase K at 37°C for 1 h, and quantified using crystal violet assay. The data are based on three independent experiments in triplicates. Asterisk (∗) denotes a significant difference between non‐treated and proteinase K treated samples (*p* < 0.05).

The masses of mono‐ and dual‐species biofilms treated with proteinase K were quantified using a crystal violet assay (Figure 3). Proteinase K treatment significantly reduced the biofilm mass of *C*. *jejuni* NCTC11168 (*p* < 0.05). Among the monospecies biofilms of the contaminating isolates, *A*. *baumannii* and *S*. *epidermidis* exhibited significant reductions (*p* < 0.05). Among the dual‐species biofilms, the dual‐species biofilm of *C. jejuni* associated with *A. baumannii* was significantly reduced following proteinase K treatment (*p* < 0.05) (Figure 3).

CLSM analysis of *A. baumannii* 9‐18 biofilm demonstrated that the biofilms were highly heterogeneous in some areas. The distribution of proteins was highly inconsistent with that of polysaccharides (Figure 4a). In contrast, the distribution of proteins was consistent with that of cells or eDNA, suggesting that extracellular proteins are highly associated with biofilm‐embedded cells. After proteinase K treatment, the intensity of proteins was greatly diminished, whereas the intensities of polysaccharides and cells were slightly decreased (Figure 4). This decrease was confirmed by quantitative measurement of the intensities of the fluorescent dyes (Table 2).

Figure 4Differential imaging analysis of protein, polysaccharide, and cells in the biofilms of (a) *A. baumannii* 9‐18 and (b) *C. jejuni* NCTC11168. Biofilms were cultivated on a microplate featuring a bottom layer made of glass‐like polymer. *A. baumannii* was isolated from a countertop for a completed menu. Biofilms were formed in MHB at 37°C for 48 h, and the bottom surface–attached biofilms were visualized using CLSM. Protein, carbohydrate, and cells were stained with FITC, tetramethylrhodamine conjugate of concanavalin A (ConA Rho), and SYTO63, respectively. Scale bars equal 50 *μ*m.

**Figure:**
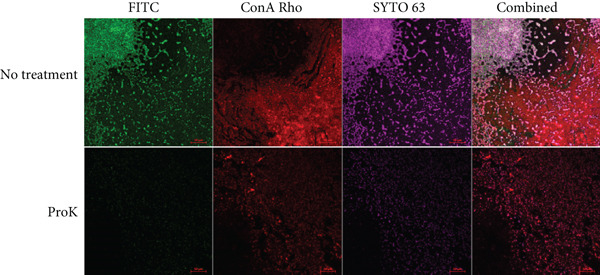
(a)

**Figure:**
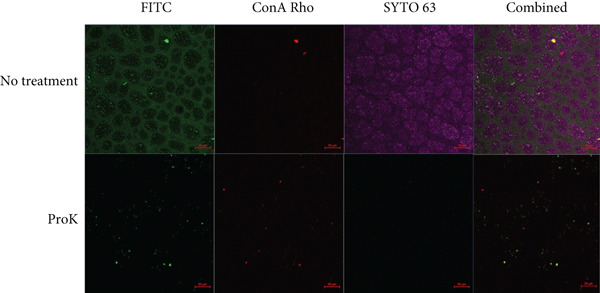
(b)

**Table 2:** Arithmetic mean intensity of fluorescent dyes staining *C. jejuni*, *A. baumannii*, and the dual‐species of biofilms.

**Strains**	**Enzymes**	**Fluorescent intensities** ^**a**^		
		**FITC**	**ConA**	**SYTO63**
*C. jejuni*	None	42.873	8.376	38.041
	DNase I	5.864	7.472	12.412
	ProK	1.028	0.726	0.007
	DNase I→ProK	0.834	0.709	0.007

*A. baumannii*	None	52.698	63.283	64.634
	DNase I	11.981	20.015	19.99
	ProK	4.15	24.437	13.109
	DNase I→ProK	6.532	11.736	22.255

*C. jejuni + A. baumannii*	None	19.177	50.492	33.535
	DNase I	13.942	15.662	22.905
	ProK	9.265	14.934	24.019
	DNase I→ProK	4.205	21.723	10.347

^a^FITC, ConA, and SYTO63 represent proteins, polysaccharides, and cells, respectively, as detected using specific fluorescent dyes optimized for each target molecule.

CLSM analysis of *C. jejuni* NCTC11168 biofilm demonstrated a somewhat distinct pattern of distribution in some areas compared to *A. baumannii* 9‐18 (Figure 4b). The relative amount of polysaccharides was very low in *C. jejuni*. The distribution of proteins was highly inconsistent with that of cells or eDNA in some cases (Figure 4b). However, it was consistent with that of cells or eDNA in other cases (data not shown). Generally, the area in which protein was dense was deficient in cells or eDNA, whereas the area in which protein was deficient was highly dense with cells or eDNA. Despite the inconsistency, the intensities of both protein and cells were greatly diminished after proteinase K treatment (Figure 4b). It may imply that proteins in *C. jejuni* biofilms may function as a cohesive structural scaffold.

**Figure 5:**
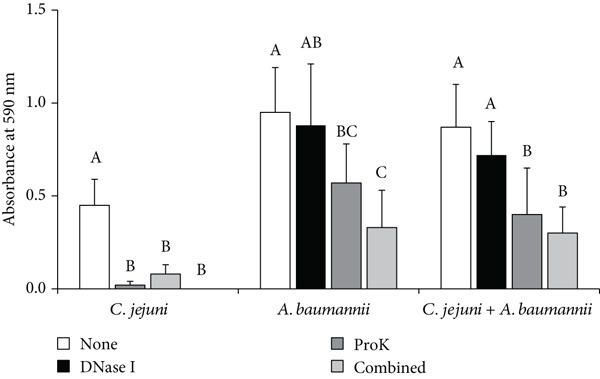
Effect of the combined biofilm treatment using DNase I and proteinase K. DNase I was applied, and the plate was then incubated at 37°C for 1 h. Following incubation, the wells were washed once with 150 *μ*L of MHB per well. Subsequently, proteinase K was added and the plate was incubated for an additional hour at 37°C.

Combined treatment with DNase I and proteinase K was studied for the dual‐species biofilm comprising *C. jejuni* and *A. baumannii* because they were both susceptible to both enzymes (Figures 5 and 6 and Table 2). DNase I treatment alone did not significantly reduce the mass of dual‐species biofilms. In contrast, proteinase K treatment alone significantly reduced the mass of the dual‐species biofilm (*p* < 0.05) (Figure 5). Notably, the combined treatment with DNase I followed by proteinase K led to a further significant reduction in *A. baumannii* mono and dual‐species biofilms (*p* < 0.05). The combined treatment reduced the biofilm mass from OD 0.95 to 0.33 and from OD 0.87 to 0.3, respectively, while either treatment reduced the biofilm mass by less than 55% (Figure 5). Overall, the combination treatment was more effective than either treatment alone.

Figure 6CLSM was utilized to visualize monospecies biofilms of *C. jejuni* and *A*. *baumannii* and dual‐species biofilm of *C. jejuni* with *A. baumannii* isolate adhered to the bottom surface (a) 2D and (b) 2.5D visualizations after treatment with extracellular matrix‐degrading enzymes. Biofilms were cultivated in MHB on a microplate featuring a glass‐like polymer base. *A. baumannii* was isolated from a kitchen countertop. The preformed biofilms were incubated with each enzyme at 37°C for 1 h, and the bottom‐attached biofilms were stained with FITC, concanavalin A conjugated with tetramethylrhodamine, and SYTO63 for proteins, polysaccharides, and cells, respectively. Scale bars equal 50 *μ*m in length.

**Figure:**
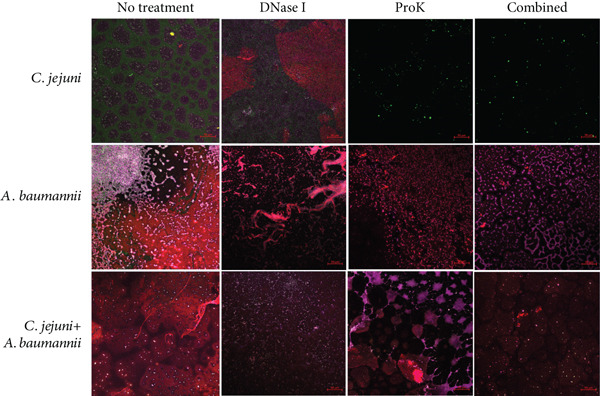
(a)

**Figure:**
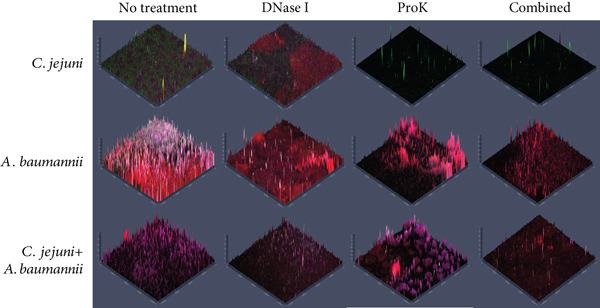
(b)

CLSM analysis generally confirmed the effect of combined treatment using DNase I and proteinase K (Figure 6). DNase I or proteinase K treatment alone showed a partial and limited reduction of the biofilms except for proteinase K treatment against *C. jejuni* biofilm. The combined treatment was generally better than a single treatment in biofilm reduction. Compared to the single treatments, the combined treatments demonstrated a clearer reduction in biofilms (Figure 6).

## 4. Discussion

Microaerobic conditions were used to form mixed‐species biofilms. We also attempted aerobic conditions that are more relevant to the food‐associated environment to form biofilms but were not successful in forming biofilms containing viable *Campylobacter*. Although our study employed microaerobic conditions for *Campylobacter* to form biofilms, we cannot exclude the possibility that multispecies biofilms can still form under aerobic conditions [31].

In this study, the viability of *C. jejuni* was significantly affected by *A. baylyi* or *S. epidermidis* in dual‐species biofilms (*p* < 0.05; Figure 1). The enhanced biofilm formation of *C. jejuni* in the presence of *S. epidermidis* is consistent with a previous study demonstrating increased biofilm formation by *C. jejuni* and *Campylobacter coli* in the presence of *S. aureus* [18]. This study demonstrated that the viable cell numbers of *C. jejuni* were highly affected by the counterpart strains in mixed‐species biofilms, which is consistent with a previous study [19].

Species and their interactions in a mixed‐species biofilm critically influence community development and shape [32]. Our study clearly shows that cohabiting microorganisms can significantly affect *Campylobacter*, and such interactions can be positive, negative, or highly species‐specific (Figure 1). Interactions among species within a biofilm can be synergistic, such as the facilitation of colonization and metabolic cooperation, or antagonistic, such as competition for nutrients and growth inhibition [32]. Such interactions have been reported in several previous studies [33–36]. For example, biofilm formation by *Aeromonas hydrophila* is inhibited by *P*. *aeruginosa* and *Pseudomonas fluorescens*, but not by *Salmonella* Typhimurium, *Listeria monocytogenes*, or *Pectobacterium carotovorum* [34]. In another study, probiotic *E. coli* inhibited biofilm formation by enterohemorrhagic *E. coli*, *S. aureus*, and *S. epidermidis*, but did not inhibit *P. aeruginosa* [33]. In another study, *Streptococcus mutans* increased biofilm formation by *Lactobacillus gasseri* and *Lactobacillus rhamnosus*, whereas it decreased biofilm formation by *Lactobacillus fermentum* [35]. In another study, a dual‐species biofilm containing *Burkholderia* significantly increased in the presence of *P. aeruginosa* PAO1, but not in the presence of *Acinetobacter calcoaceticus* or *B. subtilis* [36].

Several previous studies have demonstrated that mixed‐species biofilms provide protective effects against sanitizers or antimicrobials compared to their monospecies counterparts [15, 16, 37]. Although many studies have shown that extracellular matrix‐degrading enzymes can inhibit or degrade biofilms, relatively few studies have been conducted to test the efficacy of these enzymes against multispecies biofilms compared to monospecies biofilms [24, 38]. Our study showed that the dual‐species biofilm comprising *C. jejuni* and *A. baylyi* was resistant to DNase I treatment (Figure 2). In our previous study, such resistance to proteinase K treatment was also observed in the dual‐species biofilm of *E. coli* O157:H7 and *Acinetobacter* [37]. Sharma and Pagedar Singh found that DNase I treatment was less effective against mixed‐species biofilms comprising *P. aeruginosa*, *S. aureus*, *S*. Typhimurium, *Enterococcus faecalis*, and *Klebsiella* spp. than against monospecies biofilms [24]. In another study, *S. aureus* and *P. aeruginosa* in mixed‐species biofilms showed protective effects against cellulases and glycoside hydrolases, respectively [38].

Our data suggest that the strains of *A. baumannii* and *A. baylyi* harbor eDNA in their biofilms and that eDNA plays an important role in their structural integrity (Figure 2), which is consistent with a previous study [39]. The resistance of *B. subtilis*, *S. epidermidis*, and *K. kristinae* was consistent with that of previous studies [39–41]. Wagner et al. showed that *K. kristinae* biofilms contain small amounts of eDNA [39]. In different studies, DNase I treatment significantly inhibited biofilm formation by *B. subtilis* and *S. epidermidis*, but was ineffective against mature biofilms, which is similar to the results of our study [40, 41]. However, our results for *B. licheniformis* were inconsistent with those of previous studies [42, 43]. We speculate that strain‐to‐strain variations may exist in the biofilm structure of *B. licheniformis* such as varying amounts of eDNA or protected structures.

Proteinase K disrupts the extracellular polymeric substance (EPS) matrix by degrading its proteinaceous components, which are essential for maintaining biofilm structure and stability. Notably, the EPS of *A. baumannii* biofilms is high in proteins that play an important role in maintaining biofilm structure and integrity [44], and *A. baumannii* biofilms exhibited higher susceptibility to proteinase K treatment than DNase I in this study. This suggests that the structural integrity of *A. baumannii* biofilm was also substantially weakened by proteinase K. Compared with *C. jejuni* biofilm, this highlights the variability in biofilm composition among different bacterial species and underscores the importance of tailoring biofilm control strategies based on EPS components that play a pivotal role in biofilm integrity.

In the context of food production environments, the application of proteinase K presents a promising strategy. This potential is evidenced by its important role in disrupting biofilm formation, as the combined treatment with DNase I and proteinase K more effectively reduced the biofilm mass compared to either DNase I or proteinase K treatment alone. In addition, the ability of proteinase K to dismantle the biofilm structures by weakening the biofilm matrix and facilitating the removal of attached bacteria suggests that proteinase K could be a key tool for maintaining hygienic surfaces on food‐processing equipment and reducing bacterial regrowth after cleaning.

DNase I significantly reduced the biomass of *C. jejuni* monospecies biofilm (*p* < 0.05). However, it showed limited efficacy against *A. baumannii* monospecies biofilms and dual‐species biofilms of *C. jejuni* and *A. baumannii* (*p* > 0.05) (Figure 5). Although *C. jejuni* biofilms rely heavily on eDNA for structural stability, *A. baumannii* biofilms likely depend more on proteinaceous EPS components, making them less susceptible to DNase I treatment.

DNase I may be useful in specific areas where the biofilm matrix relies heavily on eDNA for structural integrity. However, there may be limitations in addressing biofilms with mixed or protein‐dominated matrices, requiring combined treatments to enhance their effectiveness. Therefore, combined treatment, such as the sequential application of DNase I and proteinase K, can lead to remarkable efficacy in controlling biofilms. This combined treatment had a significant impact particularly on *A. baumannii* monospecies biofilm, where biofilm biomass was substantially reduced (*p* < 0.05) (Figure 5).

This effect is attributed to a sequential mechanism of action: DNase I is applied first, targeting and weakening the eDNA‐dependent foundational structure of the biofilms. This is followed by proteinase K treatment, which more effectively degrades proteinaceous components, the major scaffolding elements of the biofilm. This approach can be especially effective in breaking down complex multicomponent biofilm matrices, offering greater biofilm disruption than standalone enzymatic treatments.

In the case of dual‐species biofilms, the combined treatment with DNase I and proteinase K showed slightly better performance than individual enzyme treatments, indicating that this outcome is characterized more as a combined effect rather than a true synergistic effect (Figure 5).

For mixed‐species biofilms, interspecies interactions and defensive mechanisms of specific bacterial species may inhibit the complete breakdown of the biofilm [45, 46]. Therefore, additional research is necessary to determine the compositional structure and interspecies dynamics of complex biofilms for broader practical applications.

Proteins and polysaccharides are the most common EPS found in bacterial biofilms. In general, the distribution of proteins is at least somewhat consistent with that of polysaccharides in bacterial biofilm EPS based on microscopic analysis [30, 47–49]. However, our study showed that the distribution of EPS in *A. baumannii* 9‐18 was highly heterogeneous, and the distribution of proteins was much more consistent with that of cells (Figure 4a). This suggests that the protein may be a major protective layer for bacterial cells of *A. baumannii* from hostile environments and is the major target to effectively inactivate biofilm‐embedded cells at the same time. Therefore, proteinase K treatment led to protein degradation, resulting in a simultaneous reduction in cells that were protected by the protein matrix (Figure 6 and Table 2).

## 5. Conclusion

DNase I and proteinase K are promising tools for enzymatic biofilm control in the food industry. The combined effects of these enzymes, particularly in mixed‐species biofilms, provide a strong foundation for the development of effective and safe sanitation protocols. Enzyme‐based cleaning solutions have the potential to revolutionize food hygiene management and enhance both food safety and operational efficiency in food manufacturing environments.

## Conflicts of Interest

The authors declare no conflicts of interest.

## Author Contributions

Formal analysis and investigation: Joo Young Lee and Joo‐Sung Kim. Writing: Joo Young Lee and Joo‐Sung Kim. Conceptualization: Joo‐Sung Kim.

## Funding

This research was funded by the Korea Food Research Institute (10.13039/501100003712) (E0210701‐05).

## Data Availability

The data that support the findings of this study are available from the corresponding author upon reasonable request.
